# A comparison of regional and general anesthesia effects on 5 year survival and cancer recurrence after transurethral resection of the bladder tumor: a retrospective analysis

**DOI:** 10.1186/s12871-016-0181-6

**Published:** 2016-03-12

**Authors:** Dale Jang, Chae Seong Lim, Yong Sup Shin, Young Kwon Ko, Sang Il Park, Seong Hyun Song, Bum June Kim

**Affiliations:** Department of Anesthesiology and Pain Medicine, Chungnam National University Hospital, Daejeon, Korea

## Abstract

**Background:**

Recent studies have reported that cancer surgeries involving regional anesthesia have better outcomes than those under general anesthesia. However, the effects of anesthetic technique have not been investigated in patients with bladder cancer. Therefore, this retrospective study was conducted to investigate which anesthetic technique results in a better bladder cancer prognosis.

**Methods:**

Sixty-one of 531 patients underwent transurethral resection of a bladder tumor under general anesthesia from 2001 to 2008 in our hospital. Patients who attended five years of follow-up and who had pathological findings of urothelial carcinoma grades I–II were enrolled. Finally, 24 patients (G group) who underwent general anesthesia and 137 (R group) who underwent regional (spinal or epidural) anesthesia were compared. Five-year survival and recurrence-free time were compared using the chi-square and *t-*tests, respectively. A logistic regression and partial correlation analysis were performed to evaluate other factors affecting survival.

**Results:**

Five-year survival was 87.5 % for general anesthesia and 96.3 % for regional (*P* = 0.099). The regression analysis showed that older age contributed to reduced survival (odds ratio = 0.85, *P* = 0.001). Regional anesthesia showed higher 5-year survival (coefficient = −0.167, *P* = 0.044) more than general anesthesia through the partial correlation analysis.

**Conclusions:**

Though partial correlation analysis show that five-year survival is higher in patients whose surgery is under regional than general anesthesia, the association was not significant in the chi-square test and logistic regression analysis. Large prospective studies are needed to determine whether the association between regional anesthesia and survival is causative.

## Background

Cancers are frightening diseases that are difficult to cure. Despite developments in chemotherapy and radiation therapy, surgical tumor excision is the main treatment. Many reports claim that anesthetic and perioperative pain control methods alter prognosis [1–4]. Although these results are tentative, several meta-analyses have supported this conclusion.

Regional anesthesia decreases surgery-induced stress and use of opioids; thus, many believe that it reduces the likelihood of cancer recurrence [1]. No retrospective studies are available on bladder cancer, even with its high frequency. Transurethral resection of the bladder tumor (TURB) is the mainstream treatment. The surgery is usually performed under regional anesthesia, but patients who have failed regional anesthesia, have a spinal deformity, or prefer not to be conscious undergo the operation under general anesthesia. We sought to determine whether mortality due to bladder cancer differs between patients whose surgeries involve general versus regional anesthesia. Therefore, this retrospective study was conducted to investigate which anesthetic approach results in a better bladder cancer prognosis.

## Methods

**Fig. 1:**
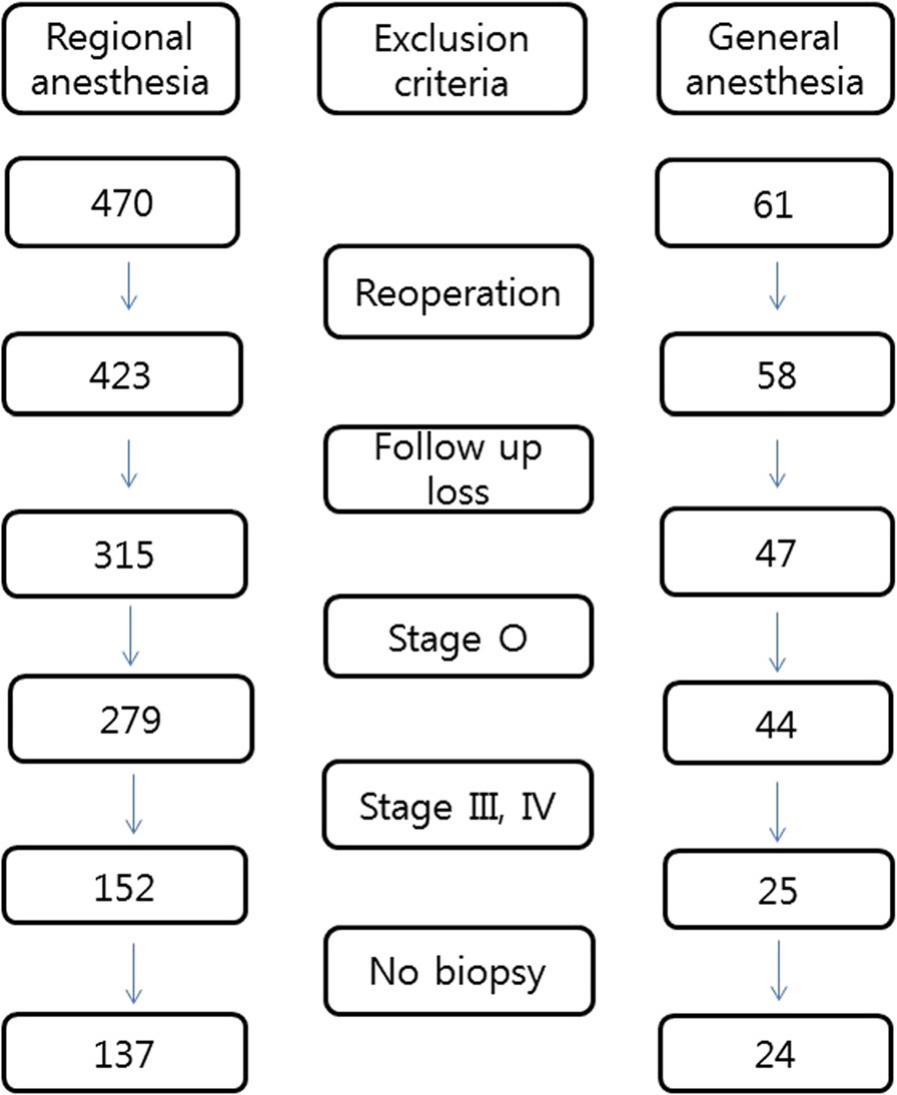
Flow chart of patient selection. Sixty-one patients underwent surgery with general anesthesia. We excluded re-operations and patients who were not followed up for five years. Patients with benign lesions and stages III and IV on biopsy specimen reports were also excluded

This retrospective study was approved by the Chungnam National University Hospital Institutional Review Board and waiver of consent was passed. TURB was performed by the same surgical and anesthesia team. Sixty-one of 531 patients underwent TURB under general anesthesia from 2001 to 2008 at our hospital. We excluded reoperations and patients who did not have five years of follow-up. We also excluded patients with benign lesions and those with biopsy specimen stages III and IV. Staging was determined based on the Cecil Textbook [5]. We excluded the case of which the cancer has grown into the layer of fatty tissue that surrounds the bladder. The consort flow chart is presented in Fig. 1. In the end, 24 patients underwent general anesthesia and were compared with 137 patients who underwent regional (spinal or epidural) anesthesia. We investigated the reasons for choosing general anesthesia. Data were collected by three residents, and statistics were processed by two independent specialists.

Propofol or etomidate was used to induce general anesthesia. Vecuronium or rocuronium was used as a neuromuscular blocker. Isoflurane or sevoflurane was used to maintain general anesthesia. Intravenous ketorolac 30 mg was administered for postoperative pain control at the end of the surgery. Lidocaine and heavy bupivacaine were used for epidural and spinal anesthesia, respectively. The number of epidural anesthesia procedures performed was 40, and the number of spinal anesthesia procedures performed was 97. Regional anesthesia patients were not treated with other analgesics for postoperative pain control.

Radiotherapy was not conducted because we included only types I and II. Intravesicular Bacillus Calmette-Guerin (BCG) was administered in most cases.

Five-year survival after surgery was analyzed by chi-square test and five-year recurrence rate and recurrence-free time after the initial surgery were analyzed with Kruskal-Wallis test. We used logistic regression analysis to determine whether BCG treatment, age, sex, smoking history, diabetes mellitus, hypertension, anesthesia type, anesthesia time, or hospital time affected survival. Additionally, a partial correlation analysis was performed. SPSS version 22.0 for Windows (IBM-SPSS Inc., Chicago, IL, USA) was used for all statistical analysis. A *p*-value < 0.05 was considered significant.

## Results

### Demographic data

Demographic data are shown in Table 1. No differences were observed between groups except for age and anesthesia time. Patients who underwent general anesthesia (67.5 ± 9.0 years) were older than those receiving regional (62.4 ± 10.8 years). Anesthesia time was 23 min longer for general than for regional anesthesia because of emergence time. Failure of regional anesthesia appeared to be the most common cause for general anesthesia, followed by previous back surgery (Table 2). No patients received a transfusion during surgery.

**Table 1:** Demographic data and *t*-test results

	General (*n* = 24)	Regional (*n* = 137)	*P* value
Age (yr)	67.5 ± 9.0	62.4 ± 10.8	0.029*
Sex (M/F)	17/7	107/30	0.438
Weight (kg)	62.0 ± 9.2	62.0 ± 9.0	0.822
Height (cm)	161.2 ± 9.6	163.1 ± 7.3	0.286
Diabetes mellitus	3	20	0.788
Hypertension	7	46	0.674
ASA (I/II/III/IV)	14/6/3/1	52/75/10/0	0.634
Anesthesia time (minutes)	77.1 ± 38.2	54.1 ± 19.7	0.000*
Hospital stay (days)	9.1 ± 5.8	7.4 ± 4.6	0.110
Pathology (I/II)	14/10	56/81	0.106
Smoking history (pack year)	13.1 ± 17.7	18.4 ± 23.0	0.113
BCG treatment patients	22	114	0.375

Values are mean ± standard deviation or numbers. No significant differences were detected, except in age and anesthesia time. *P < 0.05. *ASA* American Society of Anesthesiologists. *BCG* Bacillus Calmette-Guerin

**Table 2:** Reasons for general anesthesia

Reason	Number
Failure of regional anesthesia	8
Previous spine operation history	6
Combined operation with benign disease	4
Unrecorded	3
Patient’s need	2
Non-corporation	1
total	24

### Kruskal-Wallis test of recurrence and recurrence-free time

The recurrence rate in patients whose surgeries were performed under regional anesthesia (0.9 ± 1.4) during the five-year follow up tended to be higher than that for patients receiving general anesthesia (0.5 ± 0.8). Recurrence-free time was 45 ± 22 months in patients who underwent general anesthesia during resection and 40 ± 24 months in those receiving regional anesthesia. But recurrence rate and recurrence-free time did not show normal distribution. Therefore Kruskal-Wallis test was performed. Mean rank of recurrence rate was 73 in general group and 82 in regional group (*P* = 0.28). Mean rank of recurrence-free time 89 in general group and 80 in regional group (*P* = 0.33).

Finally, there are no statistical differences between two groups about the recurrence rate and recurrence-free time.

### Chi-square test of five-year survival

Five-year survival after initial surgery (132 of 137 patients) tended to be higher in patients who underwent regional than general anesthesia (21 of 24 patients). Five-year survival was 87.5 % for general anesthesia and 96.3 % for regional. But there was neither statistical difference (Table 3, *P* = 0.099) nor consideration about other factors.

**Table 3:** Comparison of five-year survival according to anesthesia type

	General	Regional	Total	*P* value
Survival	21	132	153	
Dead	3	5	8	
Total	24	137	161	0.099

Values are numbers. No differences were observed in five-year survival according to chi-square test

### Cause of death

Causes of total 8 deaths are as follows.

Regional anesthesia group had 5 deaths.

1. 75 years old male - Lung and bone metastasis
2. 73 years old male - Chronic renal failure and ischemic cardiomyopathy
3. 65 years old male - Bone metastasis and toxic hepatitis
4. 74 years old male - Advanced gastric cancer
5. 90 years old male - Heart failure.

General anesthesia group had 3 deaths.

1. 77 years old male - Lung and bone metastasis
2. 70 years old male - Advanced gastric cancer
3. 81 years old female - General weakness (unknown).

### Logistic regression analysis for survival

Almost variables are enrolled in logistic regression analysis for survival. ENTER method was selected because we had collected variable that could affect the survival. Odds ratio of age was 0.847 (*P* = 0.005). Older age was the main contributor to reduced five-year survival following surgery (Table 4).

**Table 4:** Logistic regression analysis for five-year survival

	Odds ratio	*P* value
Age (yr)	0.847	0.005*
Sex (M/F)	8.901	0.319
Weight (kg)	0.989	0.878
Height (cm)	1.065	0.590
Pathologic stage (I/II)	3.334	0.339
Anesthesia time (minutes)	1.016	0.541
Hospital stay (days)	0.875	0.152
Diabetes mellitus (no/yes)	0.128	0.129
Hypertension (no/yes)	0.343	0.392
ASA (I/II/III/IV)		0.517
Smoking (pack year)	0.967	0.092
BCG treatment (no/yes)	0.618	0.729
Anesthesia type (regional/general)	0.334	0.339

Many potential factors were evaluated by logistic regression analysis for five-year survival. *P < 0.05. *BCG* Bacillus Calmette-Guerin, *ASA* American Society of Anesthesiologists

### Multivariate correlation analysis

Spearman Rho correlation analysis showed that age had a significant correlation with survival (coefficient = −0.272, *P* = 0.000) and recurrence (coefficient = 0.168, *P* = 0.033). Pearson analysis showed that young patients had long recurrence free time (coefficient = −0.172, *P* = 0.029). Naturally, old patients had high ASA (coefficient = 0.216, *P* = 0.006).

General anesthesia has −0.145 of coefficient with survival (*P* = 0.066). Patients with diabetes mellitus has −0.152 of coefficient with survival (*P* = 0.055).

Female had less smoking history (coefficient = −0.156, *P* = 0.049) more than male at the time of diagnosis. Stage II patients had long hospital stay time (coefficient = 0.193, *P* = 0.014). Curiously, more diabetic patients were included in the stage II patients (coefficient = 0.179, *P* = 0.023). Anesthesia time showed positive correlation with recurrence (coefficient = 0.188, *P* = 0.017) and hospital stay (coefficient = 0.363, *P* = 0.000). Longer anesthesia time, recurrence free time was short (coefficient = −0.169, *P* = 0.032).

### Partial correlation analysis

In order to get a correct picture of the relationship between two variables, we should eliminate the influence of other variables. So we applied the partial correlation analysis to find a real factor influencing the 5-year survival. Table 5 showed the results. After controlling all other factors, age had −0.186 of coefficient and 0.024 of *P* value. Regional anesthesia showed higher 5-year survival (coefficient = −0.167, *P* = 0.044) more than general anesthesia. Recurrence free time had 0.223 of coefficient and 0.007 of *P* value. Other variables showed no significant correlation with 5-year survival.

**Table 5:** Partial correlation analysis with five-year survival

Partial correlation with survival	Control variables	coefficient	*P* value
Age (yr)	All other variables	−0.186	0.024*
Sex (M/F)		0.057	0.491
Weight (kg)		−0.008	0.922
Height (cm)		0.036	0.668
Anesthesia type (regional/general)		−0.167	0.044*
Smoking (pack year)		−0.138	0.096
Pathologic stage (I/II)		0.041	0.619
Recurrence (number during 5 years)		0.033	0.694
Recurrence free time (months)		0.223	0.007*
BCG treatment (no/yes)		−0.037	0.654
Anesthesia time (minutes)		0.129	0.120
Hospital stay (days)		−0.086	0.302
Diabetes mellitus (no/yes)		−0.081	0.332
Hypertension (no/yes)		−0.058	0.485
ASA (I/II/III/IV)		−0.031	0.707

*P < 0.05. *BCG* Bacillus Calmette-Guerin, *ASA* American Society of Anesthesiologists

## Discussion

Bladder cancers, which are usually transitional cell carcinomas [6], are often multifocal and have a high tendency to recur and progress to severe stages. Bladder cancers are classified as superficial, muscle-invasive, or metastatic. Superficial tumors are treated with the goal of preventing recurrence and progression, muscle-invasive disease is treated using surgery combined with chemotherapy and radiation therapy, and while metastatic disease cannot be eradicated, it symptoms are improved by combination chemotherapy [5]. Advances in diagnosis and treatment over the past 30 years have increased the five-year survival rate from 73 % (1974–1976) to 82 % (1998–2001) [5]. Because we excluded patients with metastases, our data show a higher five-year survival rate (95 %).

Smoking increases the prevalence of bladder cancer two to four times but had no impact on five-year survival or recurrence in our study. We only investigated smoking history as self-reported by patients; thus, it may have been inaccurately recorded. In addition, there was no record of whether or not smoking after diagnosis of bladder cancer.

Other risk factors are difficult to detect because the latency of bladder cancer can be as long as 20 years. Although cyclophosphamide also increases bladder cancer risk up to nine times [5], we were unable to investigate this factor here. According to multivariate correlation analysis, more diabetic patients had been classified the stage II than I. This result is consistent with a recent report. Fang H et al. [7] concluded that diabetes mellitus increases the risk of bladder cancer in there meta-analysis study.

Surgical resection plays an important role in cancer treatment, but cancerous cells can spread through the blood or lymph nodes [8]. As such cells’ viability and the immune response to them are affected by surgery, surgical methods can affect recurrence or mortality rates. Surgical stress, anesthetics, and opioids decrease the immune response. Surgical stress appears to be a hormonal response from the surgery itself, diminishing cell-mediated immunity [9]. Second, anesthetics hinder the immune response by affecting neutrophils, macrophages, dendritic cells, T cells, and natural killer cells [10]. Third, opioid-like agents decrease cell-mediated and humoral immunity and increase vascularity, allowing cancer cells to proliferate [1, 11].

Many studies have been conducted on the association between cancer recurrence and general anesthesia. Cummings et al. [1], in a large cohort study of 42,151 patients, reported that five-year survival is higher (adjusted hazard ratio = 0.91, *P* < 0.001) in patients who undergo epidural analgesia for a colectomy. De Oliveira et al. [2] concluded that epidural anesthesia for ovarian cancer surgery decreases the requirement for volatile agents and extends recurrence-free time. Lin et al. [3] reported that epidural anesthesia during surgery and postoperative epidural analgesia decrease the mortality rate of ovarian serous adenocarcinoma. In that study, the general anesthesia group had a hazard ratio of 1.214 (*P* = 0.043) compared with the epidural anesthesia group. Our partial correlation test showed that regional anesthesia increases 5-year survival.

However, other reports claim that regional anesthesia or analgesia has no effect on a cancer patient’s prognosis. In patients with advanced ovarian cancer (stage IIIC and IV), Lascassie et al. [12] found no benefit of epidural anesthesia during and after tumor debulking surgery to overall survival or time to recurrence. Roiss et al. [13] concluded that the oncological outcomes of 4772 patients after radical prostatectomy were not affected by adjunctive use of spinal anesthesia. In prostate cancer patients at high risk for disease progression, general anesthesia combined with epidural analgesia did not reduce the risk of cancer progression or improve survival after retro-pubic radical prostatectomy over a median observation time of 14 years [14]. As these studies involve distinct cancers, general anesthesia may have a different impact depending on the tumor, perhaps in part because of varying molecular sensitivity towards volatile agents. Our Chi-square test and logistic regression analysis showed that anesthetic technique has no significant impact on the survival.

We can not easily conclude that anesthesia affect the prognosis of bladder cancer by combining three different analysis.

Most prior studies were conducted on patients with terminal cancer. Because the factors affecting survival are complex in this subgroup, we instead surveyed patients with early cancers, which likely accounts for the strong influence of age on prognosis.

BCG is the most effective intravesical immunotherapy for treating early-stage bladder cancer. BCG is a bacterium that is related to the germ that causes tuberculosis. BCG treatment mainly affect the cells lining the inside of the bladder. For this reason, intravesical therapy is used for non-invasive or minimally invasive bladder cancers. Because more severe patients do not receive BCG, the treatment itself may not have impact on the prognosis in this study.

Conclusions from our study are limited by the small number of cases after blocking; far fewer patients received general than regional anesthesia. The patients were not randomized, and clinical care was not standardized, so selection bias and the effects of unmeasured confounding variables cannot be excluded. In addition, though anesthesia time differed between groups, TURB is a simple and short surgery, so this difference was small.

Patients receiving general anesthesia were older than those whose surgeries were performed under regional anesthesia, probably because elderly patients are more likely to have had spine surgery and degenerative anatomical changes. A study with a larger sample size is necessary, and results should be established through prospective, random allocation experiments. Other correlating factors, such as pain score, and postoperative infection rate should be examined.

## Conclusions

This is the first study to investigate the effect of anesthetic method on the prognosis of patients with non-metastatic bladder cancer. Though partial correlation analysis show that five-year survival tends to be higher in patients whose surgery is under regional than general anesthesia, the association was not significant in the chi-square test and logistic regression analysis. Effects of regional and general anesthesia on 5 year survival and cancer recurrence after transurethral resection of the bladder tumor are not certain. We cautiously suggest that there is no need to select particular anesthesia method to achieve a better prognosis in patients with non-metastatic bladder cancer undergoing TURB.
